# Eye, Ear, Nose, and Throat

**Published:** 1894-10

**Authors:** 


### *SELECTIONS AND ABSTRACTS.*

---

### EYE, EAR, NOSE, AND THROAT.

Edited by Dunbar Roy, A.B., M.D.

### The Brown-Séquard Treatment in Ocular Therapeutics.

Two articles in a recent journal on the employment of hypodermic injections of certain organic (orchitic) fluids after the manner of the late Brown-Séquard are worthy of notice, although it is questionable whether their value is any greater in ocular than in other diseases. The first is by de Wecker (*Annales d'Oculistique,* November, 1893), who draws attention to the fact that writers on ophthalmological matters, with one exception, have remained silent upon a subject that has, in late years, attracted so much attention in medical circles.

When Brown-Séquard four years ago addressed his first communication to the Biological Society, the author applied to him for some of the testicular fluid, and desired to be supplied with the proper apparatus and to be instructed in the preparation of the remedial juices. His idea was to use it in the treatment of patients afflicted by optic nerve atrophy. A large number of cases of gray atrophy, optic atrophy of central origin, and retrobulbar neurites were carefully and systematically treated and observed. The negative results obtained led de Wecker to believe that his method of preparing the organic fluids was defective, so he obtained some prepared by M. d' Arsonval, Brown-Séquard's associate, and repeated the experiments. The results were precisely the same as before: with one or two exceptions, the treatment produced no effect.

Finally de Wecker believes the injections to be entirely innocuous, and thinks they may act as a tonic and perhaps produce at the same time a moral effect upon a certain class of patients.

De Bourgon, in the same paper, gives a carefully prepared paper on the treatment of four cases of optic nerve atrophy, the histories of which are furnished in detail. The results in every case were absolutely negative. Not only that, but both earlier and subsequent employment of injections of antipyrin and the use of additional remedies by the author and others' aid, have some measurable effect upon the disease. The first case was one of advanced atrophy: Vision in right eye equals one-sixth; vision in left equals perception of light, the result of ill-defined cerebral changes. The second patient also had well-marked atrophy. Vision right eye equals one-sixtieth; vision left eye equals fingers at 3 M. In the third instance there was a secondary atrophy produced by the presence of a cerebral neoplasm. The last case was a diabetic atrophy with very low vision: R.$=\frac{1}{50}$ L.$=\frac{1}{60}$.

De Bourgon advises the use of antipyrin injections (introduced by Valude) in optic atrophy and says that two of the above mentioned patients upon whom they were used obtained greater relief than from all other measures employed in the treatment of their diseases.—*Annals of Ophthalmology and Otology*, April.

[The writer has obtained very decided results in the use of strychnia injected into the temples, a remedy which has been used for some time.—R.]

---

### INVESTIGATIONS ON THE DISINFECTION OF THE CONJUNCTIVAL SAC, WITH REMARKS ON ITS BACTERIOLOGY.

Franke has experimented on disinfection of the conjunctival sac, with simple washings with bichloride 1 : 5000; the same after previous wiping; washing with chlorine water (Schmidt-Rimpler); brushing with bichloride 1 : 2500, and washing with trichloride of iodine 1 :2000 (Pflueger).

His conclusions are as follows: (1) A conjunctiva normal in appearance may contain micro-organisms, and even such of a pathogenic nature. (2) The conjunctiva should, therefore, always be disinfected before an operation. The disadvantage of bichloride in causing parenchymatous opacity of the cornea is counterbalanced by the quick decomposition and loss of disinfecting power and smell of other agents. (3) None of these agents will sterilize the conjunctival sac with certainty. In twenty-four per cent. of cases the number of micro-organisms was apparently reduced. (4) Since the agents act only on those micro-organisms on the surface of the epithelium, it is advisable to first wipe out the sac with agents which dissolve the mucus. The use of indifferent agents, salt, boric acid solution, and the like is not advisable. (5) Micro-organisms which are not killed by disinfection do not lose thereby their virulency.—*Graefe's Archives*, XXXIX.

---

### THE THERAPEUTIC VALUE OF EUROPHEN, ALUMNOL, DIAPHTERIN, AND ANTISEPTIN IN SUPPURATION OF THE EAR.

Dr. Szenes (Budapest) tried these drugs in eighty-six cases. Diaphterin in eighteen cases caused a burning sensation which lasted five minutes. In five cases of diffuse otitis externa, as also in nine cases of chronic suppuration of the tympanic cavity, it at once caused an increase of the secretion. Antiseptin was tried on sixteen patients, and despite of its strong recommendation as an antiseptic in tubercle and syphilis, he had an unfavorable report to give about it also. Europhen proved to be an antiseptic dusting powder in suppuration of the external meatus. Alumnol was used in thirty-eight cases, causing burning sensation in those patients. The powder did not check the secretion.—*Annals of Ophthal. and Otology*.

[The writer gave alumnol a good trial with the result of being very unsatisfactory. In some cases it causes burning and increases the discharge.—R.]

---

### HYPERTROPHY OF THE LINGUAL TONSIL AS A CAUSE FOR ASTHMATIC ATTACKS.

Dr. J. Roquer Cassaden reports such a case in the *Annales des Méd. de l'Oreille et du Larynx :* A man thirty-nine years old, of a lymphatic and nervous temperament, suffered with nightly attacks of dyspnœa. There was a cough and a feeling as if a foreign body was in the throat. A drink of water relieved this condition only momentarily. The hypertrophied lingual tonsil touched the anterior surface of the epiglottis. The author looked upon this contact as a cause of the asthma, because the condition was cured by treating the hypertropied mass with the galvano-cautery.

